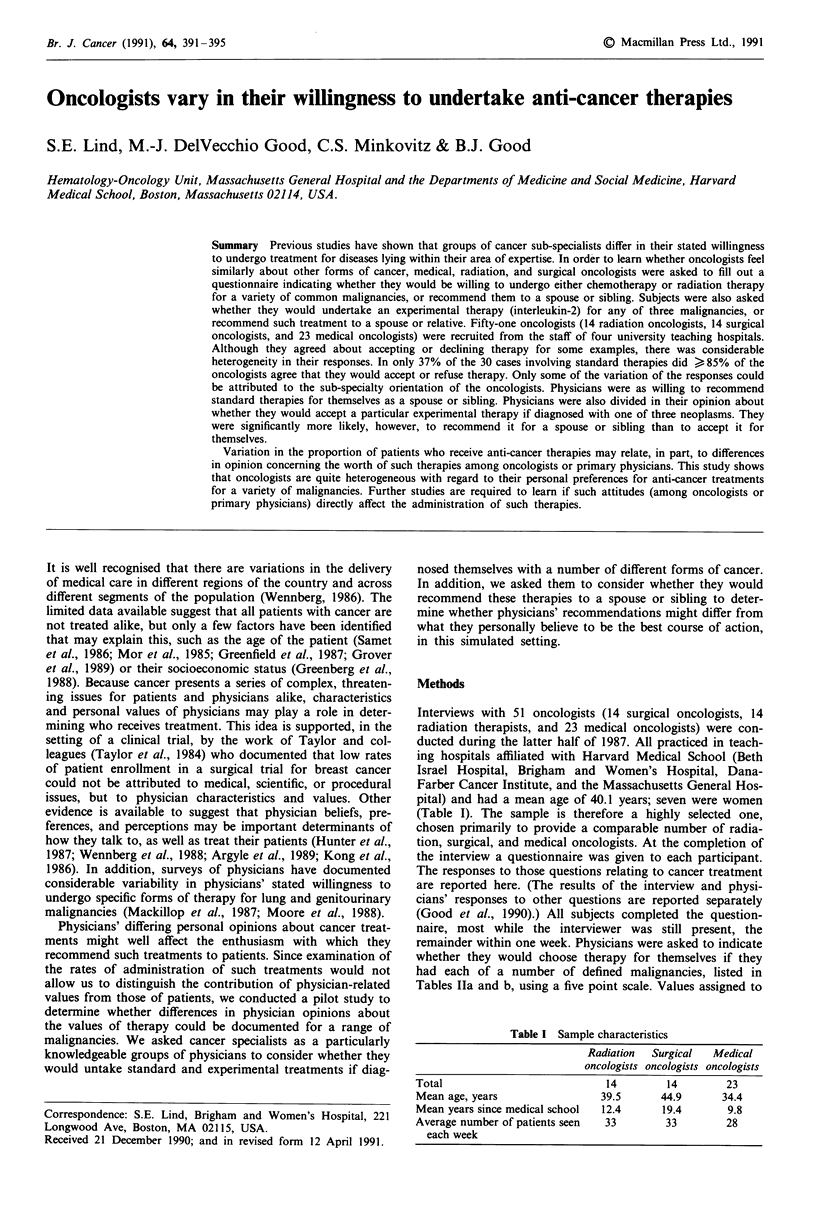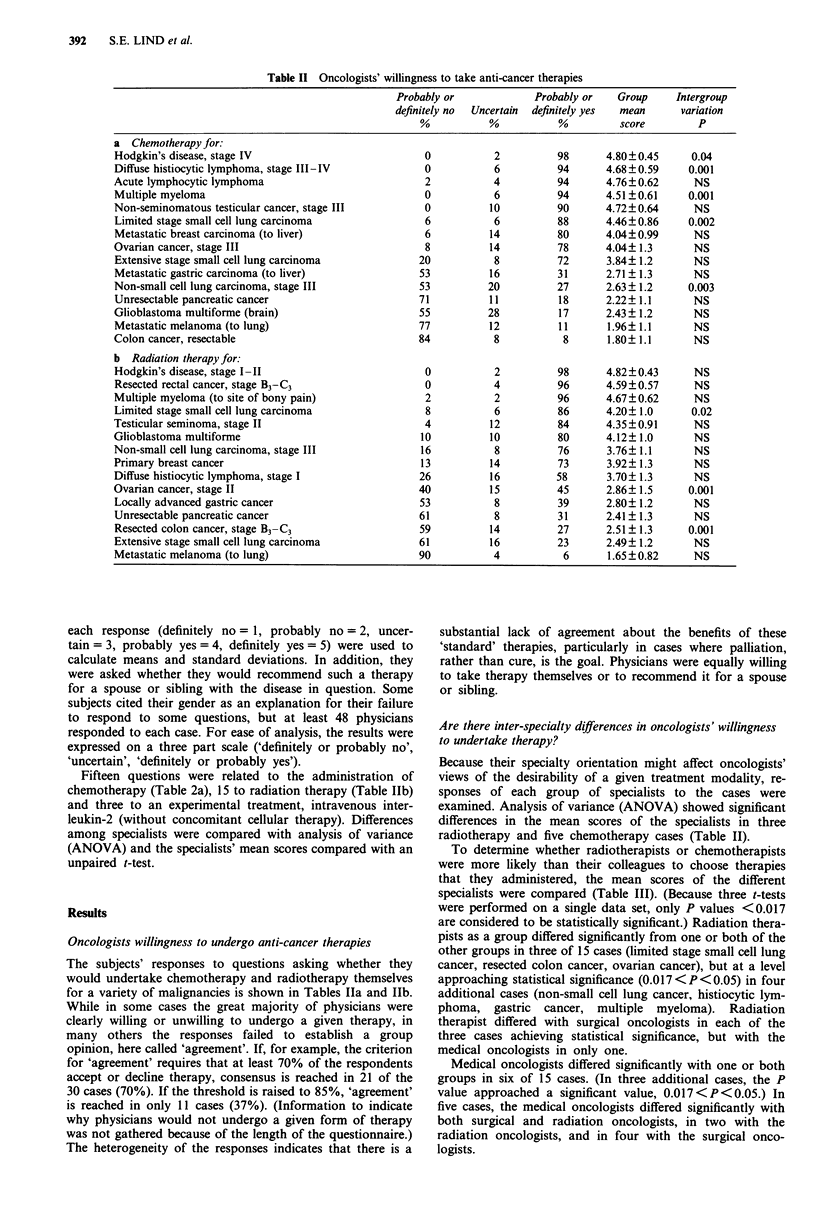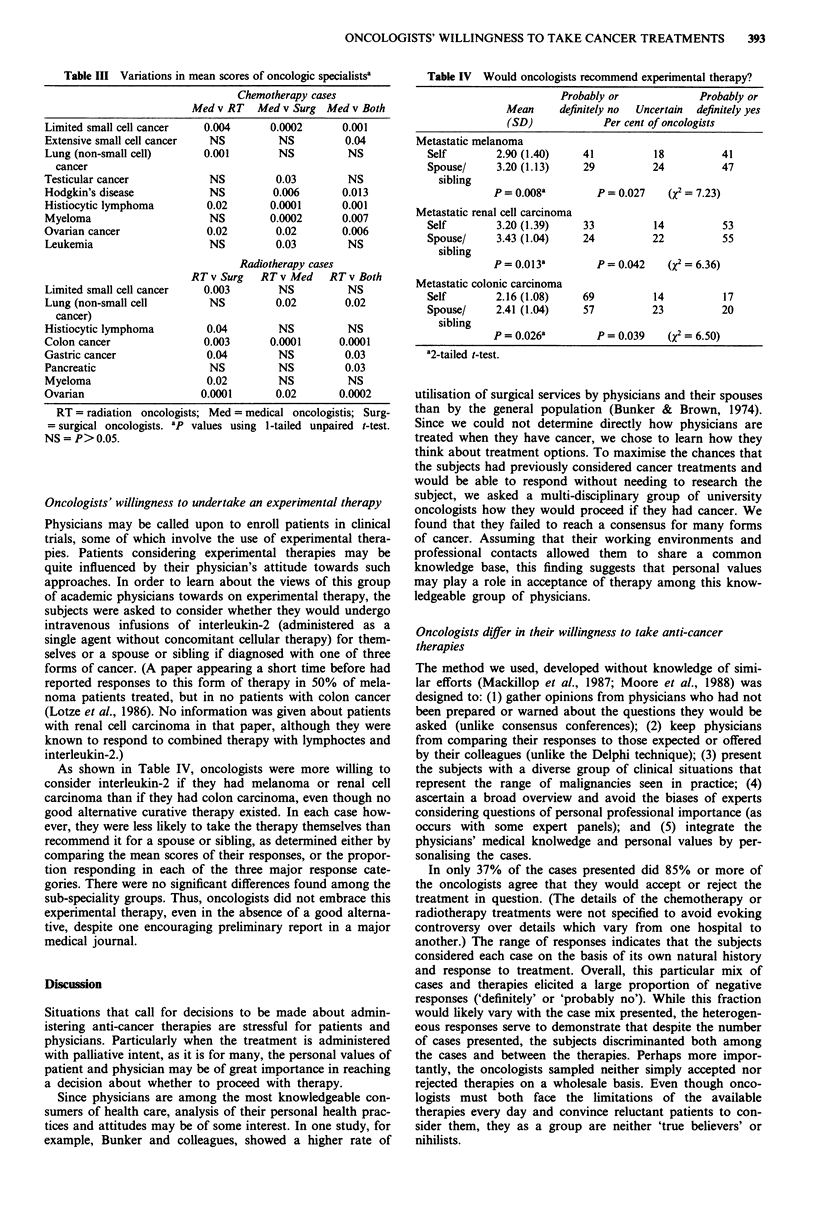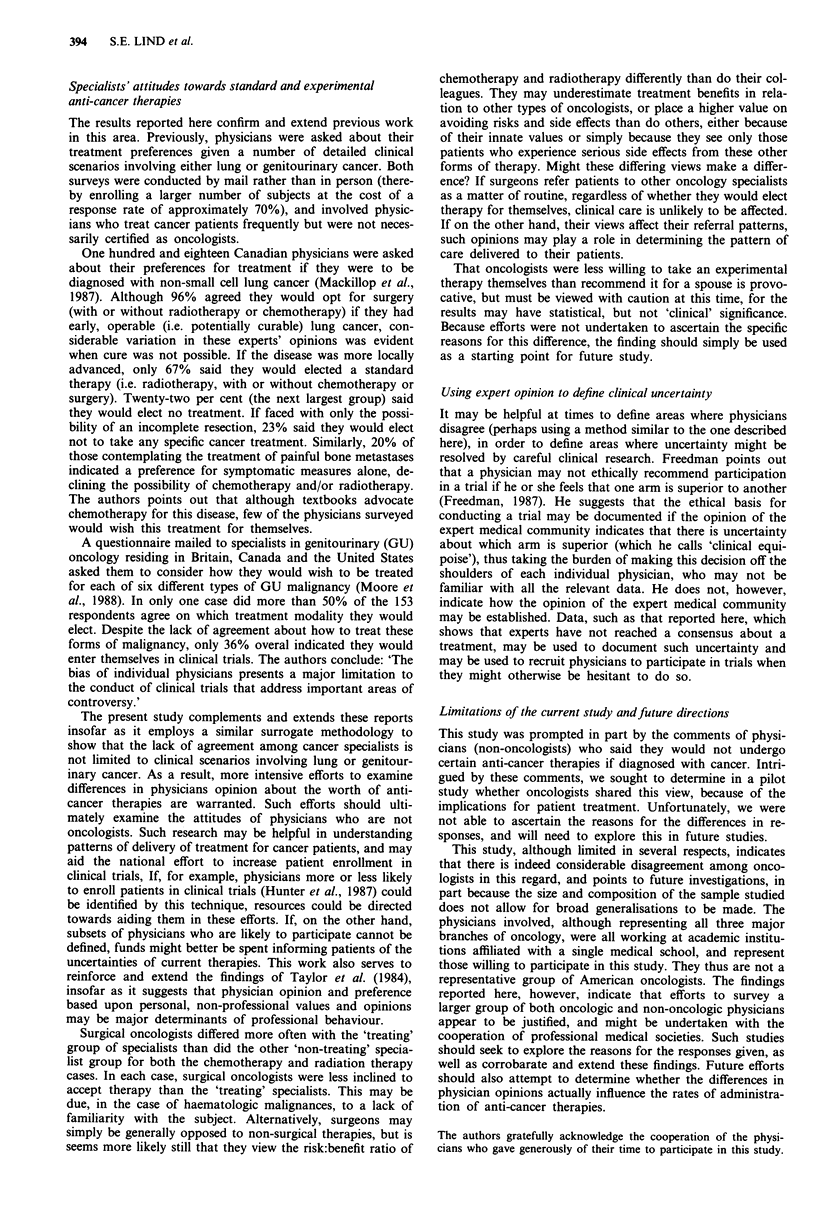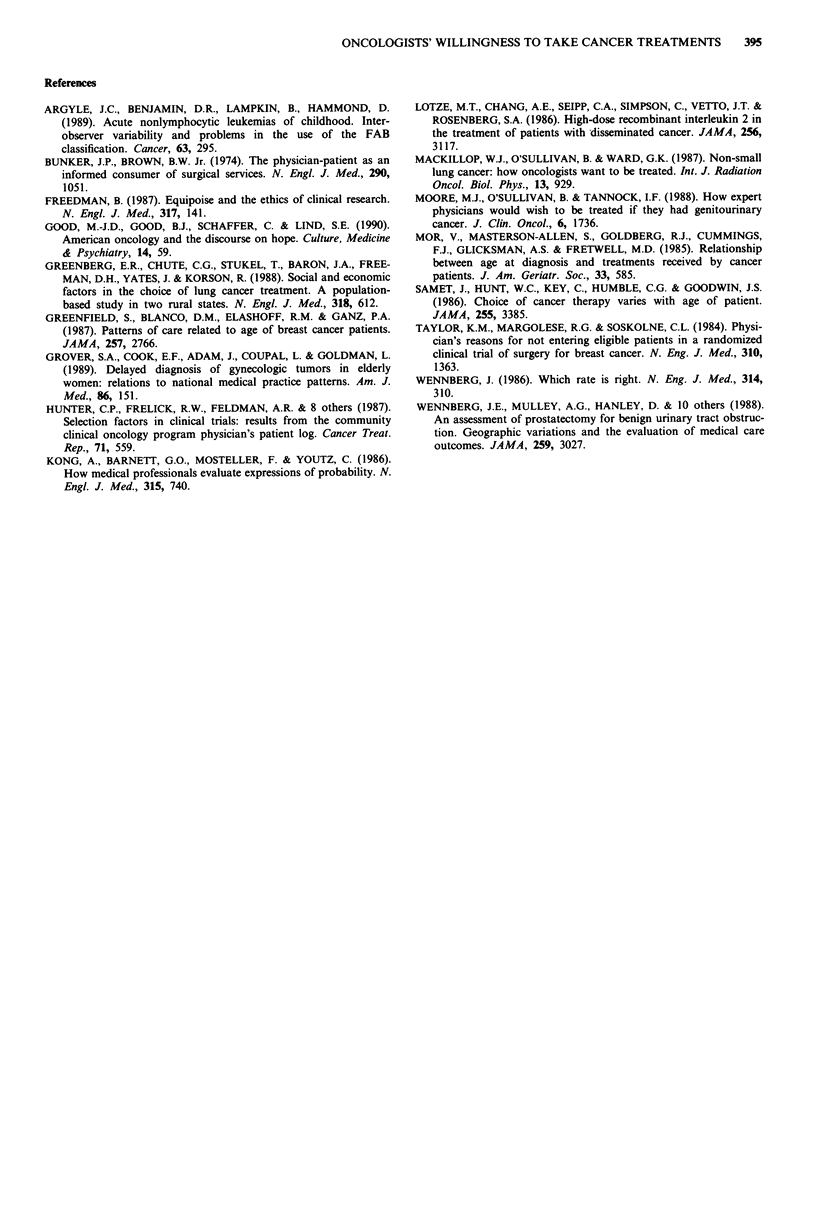# Oncologists vary in their willingness to undertake anti-cancer therapies.

**DOI:** 10.1038/bjc.1991.315

**Published:** 1991-08

**Authors:** S. E. Lind, M. J. DelVecchio Good, C. S. Minkovitz, B. J. Good

**Affiliations:** Hematology-Oncology Unit, Massachusetts General Hospital, Boston 02114.

## Abstract

Previous studies have shown that groups of cancer sub-specialists differ in their stated willingness to undergo treatment for diseases lying within their area of expertise. In order to learn whether oncologists feel similarly about other forms of cancer, medical, radiation, and surgical oncologists were asked to fill out a questionnaire indicating whether they would be willing to undergo either chemotherapy or radiation therapy for a variety of common malignancies, or recommend them to a spouse or sibling. Subjects were also asked whether they would undertake an experimental therapy (interleukin-2) for any of three malignancies, or recommend such treatment to a spouse or relative. Fifty-one oncologists (14 radiation oncologists, 14 surgical oncologists, and 23 medical oncologists) were recruited from the staff of four university teaching hospitals. Although they agreed about accepting or declining therapy for some examples, there was considerable heterogeneity in their responses. In only 37% of the 30 cases involving standard therapies did greater than or equal to 85% of the oncologists agree that they would accept or refuse therapy. Only some of the variation of the responses could be attributed to the sub-specialty orientation of the oncologists. Physicians were as willing to recommend standard therapies for themselves as a spouse or sibling. Physicians were also divided in their opinion about whether they would accept a particular experimental therapy if diagnosed with one of three neoplasms. They were significantly more likely, however, to recommend it for a spouse or sibling than to accept it for themselves. Variation in the proportion of patients who receive anti-cancer therapies may relate, in part, to differences in opinion concerning the worth of such therapies among oncologists or primary physicians. This study shows that oncologists are quite heterogeneous with regard to their personal preferences for anti-cancer treatments for a variety of malignancies. Further studies are required to learn if such attitudes (among oncologists or primary physicians) directly affect the administration of such therapies.


Br. J. Cancer (1991), 64, 391–395 © Macmillan Press Ltd., 1991

### Oncologists vary in their willingness to undertake anti-cancer therapies

S.E. Lind, M.-J. DelVecchio Good, C.S. Minkovitz & B.J. Good

*Hematology-Oncology Unit, Massachusetts General Hospital and the Departments of Medicine and Social Medicine, Harvard Medical School, Boston, Massachusetts 02114, USA.*

**Summary** Previous studies have shown that groups of cancer sub-specialists differ in their stated willingness to undergo treatment for diseases lying within their area of expertise. In order to learn whether oncologists feel similarly about other forms of cancer, medical, radiation, and surgical oncologists were asked to fill out a questionnaire indicating whether they would be willing to undergo either chemotherapy or radiation therapy for a variety of common malignancies, or recommend them to a spouse or sibling. Subjects were also asked whether they would undertake an experimental therapy (interleukin-2) for any of three malignancies, or recommend such treatment to a spouse or relative. Fifty-one oncologists (14 radiation oncologists, 14 surgical oncologists, and 23 medical oncologists) were recruited from the staff of four university teaching hospitals. Although they agreed about accepting or declining therapy for some examples, there was considerable heterogeneity in their responses. In only 37% of the 30 cases involving standard therapies did $\geqslant$85% of the oncologists agree that they would accept or refuse therapy. Only some of the variation of the responses could be attributed to the sub-specialty orientation of the oncologists. Physicians were as willing to recommend standard therapies for themselves as a spouse or sibling. Physicians were also divided in their opinion about whether they would accept a particular experimental therapy if diagnosed with one of three neoplasms. They were significantly more likely, however, to recommend it for a spouse or sibling than to accept it for themselves.

Variation in the proportion of patients who receive anti-cancer therapies may relate, in part, to differences in opinion concerning the worth of such therapies among oncologists or primary physicians. This study shows that oncologists are quite heterogeneous with regard to their personal preferences for anti-cancer treatments for a variety of malignancies. Further studies are required to learn if such attitudes (among oncologists or primary physicians) directly affect the administration of such therapies.

It is well recognised that there are variations in the delivery of medical care in different regions of the country and across different segments of the population (Wennberg, 1986). The limited data available suggest that all patients with cancer are not treated alike, but only a few factors have been identified that may explain this, such as the age of the patient (Samet *et al.*, 1986; Mor *et al.*, 1985; Greenfield *et al.*, 1987; Grover *et al.*, 1989) or their socioeconomic status (Greenberg *et al.*, 1988). Because cancer presents a series of complex, threatening issues for patients and physicians alike, characteristics and personal values of physicians may play a role in determining who receives treatment. This idea is supported, in the setting of a clinical trial, by the work of Taylor and colleagues (Taylor *et al.*, 1984) who documented that low rates of patient enrollment in a surgical trial for breast cancer could not be attributed to medical, scientific, or procedural issues, but to physician characteristics and values. Other evidence is available to suggest that physician beliefs, preferences, and perceptions may be important determinants of how they talk to, as well as treat their patients (Hunter *et al.*, 1987; Wennberg *et al.*, 1988; Argyle *et al.*, 1989; Kong *et al.*, 1986). In addition, surveys of physicians have documented considerable variability in physicians' stated willingness to undergo specific forms of therapy for lung and genitourinary malignancies (Mackillop *et al.*, 1987; Moore *et al.*, 1988).

Physicians' differing personal opinions about cancer treatments might well affect the enthusiasm with which they recommend such treatments to patients. Since examination of the rates of administration of such treatments would not allow us to distinguish the contribution of physician-related values from those of patients, we conducted a pilot study to determine whether differences in physician opinions about the values of therapy could be documented for a range of malignancies. We asked cancer specialists as a particularly knowledgeable groups of physicians to consider whether they would untake standard and experimental treatments if diagnosed themselves with a number of different forms of cancer. In addition, we asked them to consider whether they would recommend these therapies to a spouse or sibling to determine whether physicians' recommendations might differ from what they personally believe to be the best course of action, in this simulated setting.

Correspondence: S.E. Lind, Brigham and Women's Hospital, 221 Longwood Ave, Boston, MA 02115, USA.
Received 21 December 1990; and in revised form 12 April 1991.

### Methods

Interviews with 51 oncologists (14 surgical oncologists, 14 radiation therapists, and 23 medical oncologists) were conducted during the latter half of 1987. All practiced in teaching hospitals affiliated with Harvard Medical School (Beth Israel Hospital, Brigham and Women's Hospital, Dana-Farber Cancer Institute, and the Massachusetts General Hospital) and had a mean age of 40.1 years; seven were women (Table I). The sample is therefore a highly selected one, chosen primarily to provide a comparable number of radiation, surgical, and medical oncologists. At the completion of the interview a questionnaire was given to each participant. The responses to those questions relating to cancer treatment are reported here. (The results of the interview and physicians' responses to other questions are reported separately (Good *et al.*, 1990).) All subjects completed the questionnaire, most while the interviewer was still present, the remainder within one week. Physicians were asked to indicate whether they would choose therapy for themselves if they had each of a number of defined malignancies, listed in Tables IIa and b, using a five point scale. Values assigned to

**Table I** Sample characteristics

| | *Radiation oncologists* | *Surgical oncologists* | *Medical oncologists* |
|---|---|---|---|
| Total | 14 | 14 | 23 |
| Mean age, years | 39.5 | 44.9 | 34.4 |
| Mean years since medical school | 12.4 | 19.4 | 9.8 |
| Average number of patients seen each week | 33 | 33 | 28 |

**Table II** Oncologists' willingness to take anti-cancer therapies

| | *Probably or definitely no %* | *Uncertain %* | *Probably or definitely yes %* | *Group mean score* | *Intergroup variation P* |
|---|---|---|---|---|---|
| **a** *Chemotherapy for:* | | | | | |
| Hodgkin's disease, stage IV | 0 | 2 | 98 | 4.80±0.45 | 0.04 |
| Diffuse histiocytic lymphoma, stage III–IV | 0 | 6 | 94 | 4.68±0.59 | 0.001 |
| Acute lymphocytic lymphoma | 2 | 4 | 94 | 4.76±0.62 | NS |
| Multiple myeloma | 0 | 6 | 94 | 4.51±0.61 | 0.001 |
| Non-seminomatous testicular cancer, stage III | 0 | 10 | 90 | 4.72±0.64 | NS |
| Limited stage small cell lung carcinoma | 6 | 6 | 88 | 4.46±0.86 | 0.002 |
| Metastatic breast carcinoma (to liver) | 6 | 14 | 80 | 4.04±0.99 | NS |
| Ovarian cancer, stage III | 8 | 14 | 78 | 4.04±1.3 | NS |
| Extensive stage small cell lung carcinoma | 20 | 8 | 72 | 3.84±1.2 | NS |
| Metastatic gastric carcinoma (to liver) | 53 | 16 | 31 | 2.71±1.3 | NS |
| Non-small cell lung carcinoma, stage III | 53 | 20 | 27 | 2.63±1.2 | 0.003 |
| Unresectable pancreatic cancer | 71 | 11 | 18 | 2.22±1.1 | NS |
| Glioblastoma multiforme (brain) | 55 | 28 | 17 | 2.43±1.2 | NS |
| Metastatic melanoma (to lung) | 77 | 12 | 11 | 1.96±1.1 | NS |
| Colon cancer, resectable | 84 | 8 | 8 | 1.80±1.1 | NS |
| **b** *Radiation therapy for:* | | | | | |
| Hodgkin's disease, stage I–II | 0 | 2 | 98 | 4.82±0.43 | NS |
| Resected rectal cancer, stage $B_3$–$C_3$ | 0 | 4 | 96 | 4.59±0.57 | NS |
| Multiple myeloma (to site of bony pain) | 2 | 2 | 96 | 4.67±0.62 | NS |
| Limited stage small cell lung carcinoma | 8 | 6 | 86 | 4.20±1.0 | 0.02 |
| Testicular seminoma, stage II | 4 | 12 | 84 | 4.35±0.91 | NS |
| Glioblastoma multiforme | 10 | 10 | 80 | 4.12±1.0 | NS |
| Non-small cell lung carcinoma, stage III | 16 | 8 | 76 | 3.76±1.1 | NS |
| Primary breast cancer | 13 | 14 | 73 | 3.92±1.3 | NS |
| Diffuse histiocytic lymphoma, stage I | 26 | 16 | 58 | 3.70±1.3 | NS |
| Ovarian cancer, stage II | 40 | 15 | 45 | 2.86±1.5 | 0.001 |
| Locally advanced gastric cancer | 53 | 8 | 39 | 2.80±1.2 | NS |
| Unresectable pancreatic cancer | 61 | 8 | 31 | 2.41±1.3 | NS |
| Resected colon cancer, stage $B_3$–$C_3$ | 59 | 14 | 27 | 2.51±1.3 | 0.001 |
| Extensive stage small cell lung carcinoma | 61 | 16 | 23 | 2.49±1.2 | NS |
| Metastatic melanoma (to lung) | 90 | 4 | 6 | 1.65±0.82 | NS |

each response (definitely no = 1, probably no = 2, uncertain = 3, probably yes = 4, definitely yes = 5) were used to calculate means and standard deviations. In addition, they were asked whether they would recommend such a therapy for a spouse or sibling with the disease in question. Some subjects cited their gender as an explanation for their failure to respond to some questions, but at least 48 physicians responded to each case. For ease of analysis, the results were expressed on a three part scale ('definitely or probably no', 'uncertain', 'definitely or probably yes').

Fifteen questions were related to the administration of chemotherapy (Table 2a), 15 to radiation therapy (Table IIb) and three to an experimental treatment, intravenous interleukin-2 (without concomitant cellular therapy). Differences among specialists were compared with analysis of variance (ANOVA) and the specialists' mean scores compared with an unpaired $t$-test.

### Results

### *Oncologists willingness to undergo anti-cancer therapies*

The subjects' responses to questions asking whether they would undertake chemotherapy and radiotherapy themselves for a variety of malignancies is shown in Tables IIa and IIb. While in some cases the great majority of physicians were clearly willing or unwilling to undergo a given therapy, in many others the responses failed to establish a group opinion, here called 'agreement'. If, for example, the criterion for 'agreement' requires that at least 70% of the respondents accept or decline therapy, consensus is reached in 21 of the 30 cases (70%). If the threshold is raised to 85%, 'agreement' is reached in only 11 cases (37%). (Information to indicate why physicians would not undergo a given form of therapy was not gathered because of the length of the questionnaire.) The heterogeneity of the responses indicates that there is a substantial lack of agreement about the benefits of these 'standard' therapies, particularly in cases where palliation, rather than cure, is the goal. Physicians were equally willing to take therapy themselves or to recommend it for a spouse or sibling.

### *Are there inter-specialty differences in oncologists' willingness to undertake therapy?*

Because their specialty orientation might affect oncologists' views of the desirability of a given treatment modality, responses of each group of specialists to the cases were examined. Analysis of variance (ANOVA) showed significant differences in the mean scores of the specialists in three radiotherapy and five chemotherapy cases (Table II).

To determine whether radiotherapists or chemotherapists were more likely than their colleagues to choose therapies that they administered, the mean scores of the different specialists were compared (Table III). (Because three $t$-tests were performed on a single data set, only $P$ values $<0.017$ are considered to be statistically significant.) Radiation therapists as a group differed significantly from one or both of the other groups in three of 15 cases (limited stage small cell lung cancer, resected colon cancer, ovarian cancer), but at a level approaching statistical significance ($0.017<P<0.05$) in four additional cases (non-small cell lung cancer, histiocytic lymphoma, gastric cancer, multiple myeloma). Radiation therapist differed with surgical oncologists in each of the three cases achieving statistical significance, but with the medical oncologists in only one.

Medical oncologists differed significantly with one or both groups in six of 15 cases. (In three additional cases, the $P$ value approached a significant value, $0.017<P<0.05$.) In five cases, the medical oncologists differed significantly with both surgical and radiation oncologists, in two with the radiation oncologists, and in four with the surgical oncologists.

**Table III** Variations in mean scores of oncologic specialists[a]

| | *Chemotherapy cases* | | |
|---|---|---|---|
| | *Med* v *RT* | *Med* v *Surg* | *Med* v *Both* |
| Limited small cell cancer | 0.004 | 0.0002 | 0.001 |
| Extensive small cell cancer | NS | NS | 0.04 |
| Lung (non-small cell) cancer | 0.001 | NS | NS |
| Testicular cancer | NS | 0.03 | NS |
| Hodgkin's disease | NS | 0.006 | 0.013 |
| Histiocytic lymphoma | 0.02 | 0.0001 | 0.001 |
| Myeloma | NS | 0.0002 | 0.007 |
| Ovarian cancer | 0.02 | 0.02 | 0.006 |
| Leukemia | NS | 0.03 | NS |
| | *Radiotherapy cases* | | |
| | *RT* v *Surg* | *RT* v *Med* | *RT* v *Both* |
| Limited small cell cancer | 0.003 | NS | NS |
| Lung (non-small cell cancer) | NS | 0.02 | 0.02 |
| Histiocytic lymphoma | 0.04 | NS | NS |
| Colon cancer | 0.003 | 0.0001 | 0.0001 |
| Gastric cancer | 0.04 | NS | 0.03 |
| Pancreatic | NS | NS | 0.03 |
| Myeloma | 0.02 | NS | NS |
| Ovarian | 0.0001 | 0.02 | 0.0002 |

RT = radiation oncologists; Med = medical oncologistis; Surg- = surgical oncologists. [a]*P* values using 1-tailed unpaired *t*-test. NS = $P > 0.05$.

**Table IV** Would oncologists recommend experimental therapy?

| | Mean (SD) | Probably or definitely no | Uncertain | Probably or definitely yes |
|---|---|---|---|---|
| | | *Per cent of oncologists* | | |
| Metastatic melanoma | | | | |
| Self | 2.90 (1.40) | 41 | 18 | 41 |
| Spouse/ sibling | 3.20 (1.13) | 29 | 24 | 47 |
| | $P = 0.008$[a] | $P = 0.027$ | ($\chi^2 = 7.23$) | |
| Metastatic renal cell carcinoma | | | | |
| Self | 3.20 (1.39) | 33 | 14 | 53 |
| Spouse/ sibling | 3.43 (1.04) | 24 | 22 | 55 |
| | $P = 0.013$[a] | $P = 0.042$ | ($\chi^2 = 6.36$) | |
| Metastatic colonic carcinoma | | | | |
| Self | 2.16 (1.08) | 69 | 14 | 17 |
| Spouse/ sibling | 2.41 (1.04) | 57 | 23 | 20 |
| | $P = 0.026$[a] | $P = 0.039$ | ($\chi^2 = 6.50$) | |

[a]2-tailed *t*-test.

*Oncologists' willingness to undertake an experimental therapy*

Physicians may be called upon to enroll patients in clinical trials, some of which involve the use of experimental therapies. Patients considering experimental therapies may be quite influenced by their physician's attitude towards such approaches. In order to learn about the views of this group of academic physicians towards on experimental therapy, the subjects were asked to consider whether they would undergo intravenous infusions of interleukin-2 (administered as a single agent without concomitant cellular therapy) for themselves or a spouse or sibling if diagnosed with one of three forms of cancer. (A paper appearing a short time before had reported responses to this form of therapy in 50% of melanoma patients treated, but in no patients with colon cancer (Lotze *et al.*, 1986). No information was given about patients with renal cell carcinoma in that paper, although they were known to respond to combined therapy with lymphoctes and interleukin-2.)

As shown in Table IV, oncologists were more willing to consider interleukin-2 if they had melanoma or renal cell carcinoma than if they had colon carcinoma, even though no good alternative curative therapy existed. In each case however, they were less likely to take the therapy themselves than recommend it for a spouse or sibling, as determined either by comparing the mean scores of their responses, or the proportion responding in each of the three major response categories. There were no significant differences found among the sub-speciality groups. Thus, oncologists did not embrace this experimental therapy, even in the absence of a good alternative, despite one encouraging preliminary report in a major medical journal.

### Discussion

Situations that call for decisions to be made about administering anti-cancer therapies are stressful for patients and physicians. Particularly when the treatment is administered with palliative intent, as it is for many, the personal values of patient and physician may be of great importance in reaching a decision about whether to proceed with therapy.

Since physicians are among the most knowledgeable consumers of health care, analysis of their personal health practices and attitudes may be of some interest. In one study, for example, Bunker and colleagues, showed a higher rate of utilisation of surgical services by physicians and their spouses than by the general population (Bunker & Brown, 1974). Since we could not determine directly how physicians are treated when they have cancer, we chose to learn how they think about treatment options. To maximise the chances that the subjects had previously considered cancer treatments and would be able to respond without needing to research the subject, we asked a multi-disciplinary group of university oncologists how they would proceed if they had cancer. We found that they failed to reach a consensus for many forms of cancer. Assuming that their working environments and professional contacts allowed them to share a common knowledge base, this finding suggests that personal values may play a role in acceptance of therapy among this knowledgeable group of physicians.

*Oncologists differ in their willingness to take anti-cancer therapies*

The method we used, developed without knowledge of similar efforts (Mackillop *et al.*, 1987; Moore *et al.*, 1988) was designed to: (1) gather opinions from physicians who had not been prepared or warned about the questions they would be asked (unlike consensus conferences); (2) keep physicians from comparing their responses to those expected or offered by their colleagues (unlike the Delphi technique); (3) present the subjects with a diverse group of clinical situations that represent the range of malignancies seen in practice; (4) ascertain a broad overview and avoid the biases of experts considering questions of personal professional importance (as occurs with some expert panels); and (5) integrate the physicians' medical knolwedge and personal values by personalising the cases.

In only 37% of the cases presented did 85% or more of the oncologists agree that they would accept or reject the treatment in question. (The details of the chemotherapy or radiotherapy treatments were not specified to avoid evoking controversy over details which vary from one hospital to another.) The range of responses indicates that the subjects considered each case on the basis of its own natural history and response to treatment. Overall, this particular mix of cases and therapies elicited a large proportion of negative responses ('definitely' or 'probably no'). While this fraction would likely vary with the case mix presented, the heterogeneous responses serve to demonstrate that despite the number of cases presented, the subjects discriminanted both among the cases and between the therapies. Perhaps more importantly, the oncologists sampled neither simply accepted nor rejected therapies on a wholesale basis. Even though oncologists must both face the limitations of the available therapies every day and convince reluctant patients to consider them, they as a group are neither 'true believers' or nihilists.

*Specialists' attitudes towards standard and experimental anti-cancer therapies*

The results reported here confirm and extend previous work in this area. Previously, physicians were asked about their treatment preferences given a number of detailed clinical scenarios involving either lung or genitourinary cancer. Both surveys were conducted by mail rather than in person (thereby enrolling a larger number of subjects at the cost of a response rate of approximately 70%), and involved physicians who treat cancer patients frequently but were not necessarily certified as oncologists.

One hundred and eighteen Canadian physicians were asked about their preferences for treatment if they were to be diagnosed with non-small cell lung cancer (Mackillop *et al.*, 1987). Although 96% agreed they would opt for surgery (with or without radiotherapy or chemotherapy) if they had early, operable (i.e. potentially curable) lung cancer, considerable variation in these experts' opinions was evident when cure was not possible. If the disease was more locally advanced, only 67% said they would elected a standard therapy (i.e. radiotherapy, with or without chemotherapy or surgery). Twenty-two per cent (the next largest group) said they would elect no treatment. If faced with only the possibility of an incomplete resection, 23% said they would elect not to take any specific cancer treatment. Similarly, 20% of those contemplating the treatment of painful bone metastases indicated a preference for symptomatic measures alone, declining the possibility of chemotherapy and/or radiotherapy. The authors points out that although textbooks advocate chemotherapy for this disease, few of the physicians surveyed would wish this treatment for themselves.

A questionnaire mailed to specialists in genitourinary (GU) oncology residing in Britain, Canada and the United States asked them to consider how they would wish to be treated for each of six different types of GU malignancy (Moore *et al.*, 1988). In only one case did more than 50% of the 153 respondents agree on which treatment modality they would elect. Despite the lack of agreement about how to treat these forms of malignancy, only 36% overal indicated they would enter themselves in clinical trials. The authors conclude: 'The bias of individual physicians presents a major limitation to the conduct of clinical trials that address important areas of controversy.'

The present study complements and extends these reports insofar as it employs a similar surrogate methodology to show that the lack of agreement among cancer specialists is not limited to clinical scenarios involving lung or genitourinary cancer. As a result, more intensive efforts to examine differences in physicians opinion about the worth of anti-cancer therapies are warranted. Such efforts should ultimately examine the attitudes of physicians who are not oncologists. Such research may be helpful in understanding patterns of delivery of treatment for cancer patients, and may aid the national effort to increase patient enrollment in clinical trials, If, for example, physicians more or less likely to enroll patients in clinical trials (Hunter *et al.*, 1987) could be identified by this technique, resources could be directed towards aiding them in these efforts. If, on the other hand, subsets of physicians who are likely to participate cannot be defined, funds might better be spent informing patients of the uncertainties of current therapies. This work also serves to reinforce and extend the findings of Taylor *et al.* (1984), insofar as it suggests that physician opinion and preference based upon personal, non-professional values and opinions may be major determinants of professional behaviour.

Surgical oncologists differed more often with the 'treating' group of specialists than did the other 'non-treating' specialist group for both the chemotherapy and radiation therapy cases. In each case, surgical oncologists were less inclined to accept therapy than the 'treating' specialists. This may be due, in the case of haematologic malignances, to a lack of familiarity with the subject. Alternatively, surgeons may simply be generally opposed to non-surgical therapies, but is seems more likely still that they view the risk:benefit ratio of chemotherapy and radiotherapy differently than do their colleagues. They may underestimate treatment benefits in relation to other types of oncologists, or place a higher value on avoiding risks and side effects than do others, either because of their innate values or simply because they see only those patients who experience serious side effects from these other forms of therapy. Might these differing views make a difference? If surgeons refer patients to other oncology specialists as a matter of routine, regardless of whether they would elect therapy for themselves, clinical care is unlikely to be affected. If on the other hand, their views affect their referral patterns, such opinions may play a role in determining the pattern of care delivered to their patients.

That oncologists were less willing to take an experimental therapy themselves than recommend it for a spouse is provocative, but must be viewed with caution at this time, for the results may have statistical, but not 'clinical' significance. Because efforts were not undertaken to ascertain the specific reasons for this difference, the finding should simply be used as a starting point for future study.

*Using expert opinion to define clinical uncertainty*

It may be helpful at times to define areas where physicians disagree (perhaps using a method similar to the one described here), in order to define areas where uncertainty might be resolved by careful clinical research. Freedman points out that a physician may not ethically recommend participation in a trial if he or she feels that one arm is superior to another (Freedman, 1987). He suggests that the ethical basis for conducting a trial may be documented if the opinion of the expert medical community indicates that there is uncertainty about which arm is superior (which he calls 'clinical equipoise'), thus taking the burden of making this decision off the shoulders of each individual physician, who may not be familiar with all the relevant data. He does not, however, indicate how the opinion of the expert medical community may be established. Data, such as that reported here, which shows that experts have not reached a consensus about a treatment, may be used to document such uncertainty and may be used to recruit physicians to participate in trials when they might otherwise be hesitant to do so.

*Limitations of the current study and future directions*

This study was prompted in part by the comments of physicians (non-oncologists) who said they would not undergo certain anti-cancer therapies if diagnosed with cancer. Intrigued by these comments, we sought to determine in a pilot study whether oncologists shared this view, because of the implications for patient treatment. Unfortunately, we were not able to ascertain the reasons for the differences in responses, and will need to explore this in future studies.

This study, although limited in several respects, indicates that there is indeed considerable disagreement among oncologists in this regard, and points to future investigations, in part because the size and composition of the sample studied does not allow for broad generalisations to be made. The physicians involved, although representing all three major branches of oncology, were all working at academic institutions affiliated with a single medical school, and represent those willing to participate in this study. They thus are not a representative group of American oncologists. The findings reported here, however, indicate that efforts to survey a larger group of both oncologic and non-oncologic physicians appear to be justified, and might be undertaken with the cooperation of professional medical societies. Such studies should seek to explore the reasons for the responses given, as well as corrobarate and extend these findings. Future efforts should also attempt to determine whether the differences in physician opinions actually influence the rates of administration of anti-cancer therapies.

The authors gratefully acknowledge the cooperation of the physicians who gave generously of their time to participate in this study.

### References

ARGYLE, J.C., BENJAMIN, D.R., LAMPKIN, B., HAMMOND, D. (1989). Acute nonlymphocytic leukemias of childhood. Interobserver variability and problems in the use of the FAB classification. *Cancer*, **63**, 295.

BUNKER, J.P., BROWN, B.W. Jr. (1974). The physician-patient as an informed consumer of surgical services. *N. Engl. J. Med.*, **290**, 1051.

FREEDMAN, B. (1987). Equipoise and the ethics of clinical research. *N. Engl. J. Med.*, **317**, 141.

GOOD, M.-J.D., GOOD, B.J., SCHAFFER, C. & LIND, S.E. (1990). American oncology and the discourse on hope. *Culture, Medicine & Psychiatry*, **14**, 59.

GREENBERG, E.R., CHUTE, C.G., STUKEL, T., BARON, J.A., FREEMAN, D.H., YATES, J. & KORSON, R. (1988). Social and economic factors in the choice of lung cancer treatment. A population-based study in two rural states. *N. Engl. J. Med.*, **318**, 612.

GREENFIELD, S., BLANCO, D.M., ELASHOFF, R.M. & GANZ, P.A. (1987). Patterns of care related to age of breast cancer patients. *JAMA*, **257**, 2766.

GROVER, S.A., COOK, E.F., ADAM, J., COUPAL, L. & GOLDMAN, L. (1989). Delayed diagnosis of gynecologic tumors in elderly women: relations to national medical practice patterns. *Am. J. Med.*, **86**, 151.

HUNTER, C.P., FRELICK, R.W., FELDMAN, A.R. & 8 others (1987). Selection factors in clinical trials: results from the community clinical oncology program physician's patient log. *Cancer Treat. Rep.*, **71**, 559.

KONG, A., BARNETT, G.O., MOSTELLER, F. & YOUTZ, C. (1986). How medical professionals evaluate expressions of probability. *N. Engl. J. Med.*, **315**, 740.

LOTZE, M.T., CHANG, A.E., SEIPP, C.A., SIMPSON, C., VETTO, J.T. & ROSENBERG, S.A. (1986). High-dose recombinant interleukin 2 in the treatment of patients with disseminated cancer. *JAMA*, **256**, 3117.

MACKILLOP, W.J., O'SULLIVAN, B. & WARD, G.K. (1987). Non-small lung cancer: how oncologists want to be treated. *Int. J. Radiation Oncol. Biol. Phys.*, **13**, 929.

MOORE, M.J., O'SULLIVAN, B. & TANNOCK, I.F. (1988). How expert physicians would wish to be treated if they had genitourinary cancer. *J. Clin. Oncol.*, **6**, 1736.

MOR, V., MASTERSON-ALLEN, S., GOLDBERG, R.J., CUMMINGS, F.J., GLICKSMAN, A.S. & FRETWELL, M.D. (1985). Relationship between age at diagnosis and treatments received by cancer patients. *J. Am. Geriatr. Soc.*, **33**, 585.

SAMET, J., HUNT, W.C., KEY, C., HUMBLE, C.G. & GOODWIN, J.S. (1986). Choice of cancer therapy varies with age of patient. *JAMA*, **255**, 3385.

TAYLOR, K.M., MARGOLESE, R.G. & SOSKOLNE, C.L. (1984). Physician's reasons for not entering eligible patients in a randomized clinical trial of surgery for breast cancer. *N. Eng. J. Med.*, **310**, 1363.

WENNBERG, J. (1986). Which rate is right. *N. Eng. J. Med.*, **314**, 310.

WENNBERG, J.E., MULLEY, A.G., HANLEY, D. & 10 others (1988). An assessment of prostatectomy for benign urinary tract obstruction. Geographic variations and the evaluation of medical care outcomes. *JAMA*, **259**, 3027.